# Current Challenges in Bone Biology

**DOI:** 10.4172/2379-1764.1000132

**Published:** 2015-09-09

**Authors:** Hemanth Akkiraju, Anja Nohe

**Affiliations:** University of Delaware, Newark, 19716, Delaware

The bone is a very important organ that supports is one of the many bodily functions. It has very diverse functions from the general support of the human body to energy regulation and balance [1]. Bones are formed during the development either through intramembranous (flat bones) ossification or endochondral ossification (long bones) [2–4]. The human body is composed of over 270 bones at birth and fuse to become 206 in totals at adulthood that all hold crucial functions. Bones consisting of mineralized bone tissue also consists of bone marrow, nerves and blood vessels and the communication between cells in the tissues is tightly regulated by the bone environment. Bone is an active tissue that is maintained by bone cells such as osteoblasts that form bone and osteoclasts that resorb bone [5]. Additionally, within the collagen and mineral matrix osteocytes are also embedded and respond to the bone environment [6,7]. The balance between these cells is necessary to maintain bone function. Bone research is considerably a challenging field due to the intricately dense structural composition of the bone morphology. While other tissues can be easily processed and prepared for experiments, working with bone is difficult [8,9]. Due to its composition of collagen fibers and minerals, bone creates a very dense structure, in which the bone cells are embedded [10]. Therefore, studying intracellular dynamics of the bone cells embedded within the mineralized tissue has proven to be challenging a task.

Imaging cells at subcellular level within the bone environment is very challenging. Conventional intracellular studies are performed on decalcified thin tissue slices embedded in paraffin [9]. However, this kind of bone sample preparation can lead to significant changes in bones biochemical properties of antigenicity and to its mineral structure [9]. Alternatively, non-decalcified bone samples can be processed in resin based polymers and be labeled fluorescently for target proteins [8,11,12]. However, current methods are tedious and very limited. Most approaches used to image cells within the bone such as MRI, Micro-CT or Ultrasound can image bone structure and recently cells, however these techniques are limited by their low resolution at the cellular level [13].

Tissues embedded within the bone itself such as the bone marrow niche and blood vessels are easier to analyze. For example real time imaging of the bone marrow niche within bone was recently achieved [14,15]. Similarly, fluorescent imaging of cells within the bone marrow niche was also achieved [16]. However, determining the localization of cell types and protein expression dynamics of single cells within the bone is still very difficult. Recent advancements in imaging techniques allows for the identification of osteocytes embedded in the bone matrix [17]. However, more research is needed to identify intracellular protein activities of the cell bodies embedded within mineralized matrix.

Alternatively, researchers study cell dynamics in ex vivo models. Several ex vivo models of bone are developed to study cellular dynamics of bone [18–21]. These novels ex vivo bone cultures are proposed for studying inflammatory responses, cancer metastasis, and also Zetos bone bioreactor used to study bone growth utilizing mechanosensitive loading are a few good examples [19,22,23]. These ex vivo models can overcome many ethical and clinical issues that are otherwise not permissive for animal or human trials. Such model systems also allow for the imaging of bone cells more feasible [24]. One model for example uses trabecular bone samples and replaces the cells in the 3D architecture with live cells. This allows for a controlled environment within the ex vivo model structure to study bone cell function [25]. Other researchers try to recreate specific bone environments for cells by using hydrogels or porous microspheres to support 3D growth of cells [26,27]. As these data show the cells in a 3D environment show often completely different cellular dynamics as compared to their 2D cultures [28]. Although mimicking the tissue environment through ex vivo model systems makes a significant breakthrough in testing cellular responses but it is still hard to replicate the exact environmental processes.

To better understand bone function we desperately need the development of new protocols and methods to drive bone research. This is especially important to address the cause of bone diseases and their possible treatment options. Bone diseases such as osteoporosis tremendously impact on the quality of life of individuals. Musculoskeletal diseases affect one out of every two people in the United States age 18 and over, and nearly three out of four age 65 and over [29]. However, in order to develop treatments one needs to understand the basic cellular mechanisms first.
